# Optimization of Hot-Press Sintering for Cu^2+^-Sn^4+^ Co-Doped YIG Ferrites: Microstructure, Dielectric Properties, and Magnetic Properties

**DOI:** 10.3390/ma18163749

**Published:** 2025-08-11

**Authors:** Yuhao Sun, Xin Meng, Jiawen Wu, Renhao Li, Xinrong Ren, Jia Gu, Xiaoyuan Zhou, Yanhui Wu, Hui Zheng

**Affiliations:** 1 Laboratory for Nanoelectronics and NanoDevices, Department of Electronics Science and Technology, Hangzhou Dianzi University, Hangzhou 310018, China; 19357380661@163.com (Y.S.); 23010430@hdu.edu.cn (X.M.); 23040505@hdu.edu.cn (J.W.); 23040937@hdu.edu.cn (R.L.); 18868767621@163.com (X.R.); 13754237423@163.com (J.G.); zxy1852584643@126.com (X.Z.); 2 Ninth Institute, China Electronics Technology Group, Mianyang 621000, China; brillice153wyh@126.com

**Keywords:** yttrium iron garnet, hot-press sintering, magnetic property, ferromagnetic resonance linewidth, microstructure

## Abstract

Yttrium iron garnet (YIG), as a core material in microwave devices, remains a key focus in materials science for performance optimization. In this study, Y_3_Fe_4.8_Cu_0.1_Sn_0.1_O_12_ samples were prepared via the solid-phase method with the co-doping of low-magnetic-anisotropy Cu^2+^ and Sn^4+^, combined with hot-press sintering under different conditions. Systematic analyses revealed that hot-press sintering optimized the microstructure, reduced porosity, and improved the compactness to 5.60 g/cm^3^. The sample hot-pressed sintered at 1200 °C achieved a maximum ***ε***′ of 34, the lowest dielectric loss and a minimal FMR linewidth of 21 Oe, thus holding great potential for applications in high-frequency microwave devices requiring low loss and high integration. This work provides a viable approach to regulating the microstructure, dielectric properties, and magnetic properties of YIG ferrites.

## 1. Introduction

Yttrium iron garnet (YIG, Y_3_Fe_5_O_12_) is extensively utilized in microwave devices such as circulators and isolators due to its narrow ferromagnetic resonance (FMR) linewidth, controllable dielectric constant, and appropriate saturation magnetization [1,2,3]. With the swift progression of contemporary communication technology, YIG materials are required to meet the development trends of integration, low loss, and high performance [4,5]. Therefore, improving the performance of YIG materials has become a key research direction in the current field of materials science [6].

YIG has a garnet-type cubic crystal structure with the space group Ia-3, and its single unit cell is composed of three sublattices: dodecahedron, octahedron, and tetrahedron [7,8]. Current studies on the performance regulation of YIG materials mainly focus on the effects of ion doping on its lattice structure, ion occupation, and micro morphology [9]. Its dielectric properties depend on the polarization effect, and the polarization intensity is related to factors such as its material composition (including ion radius), structure, porosity, and temperature and frequency. The magnetism of YIG materials mainly originates from the Fe-O-Fe superexchange interaction between Fe^3+^ ions, and this interaction is affected by the crystal structure. In addition, since Y^3+^ ions are non-magnetic with a zero magnetic moment, the magnetism can also be regulated by substituting Y^3+^ ions with magnetic ions. The ferromagnetic resonance linewidth of YIG consists of the intrinsic linewidth, anisotropic field broadening, porosity broadening, etc., so the ferromagnetic resonance linewidth can be regulated by changing the magnetocrystalline anisotropy and material porosity. Additionally, the content of Fe^2+^ ions plays an important role in dielectric loss and the ferromagnetic resonance linewidth. The content of Fe^2+^ can be adjusted by doping high-valence or low-valence metal ions, thereby regulating the magnetic and dielectric properties [10]. Therefore, ion doping strategies usually include introducing metal ions with low magnetocrystalline anisotropy, rare earth cations with larger radii, and low-melting-point metal ions [11,12].

Hot-press sintering is a sintering technology that densifies powder materials under high-temperature and high-pressure conditions [13]. Compared with traditional pressureless sintering, the hot-press sintering process can effectively optimize the microstructure of materials, thereby enhancing the density and mechanical strength [14,15,16]. Its application in YIG materials has the following advantages [17]: On the one hand, hot-press sintering can significantly reduce the porosity of YIG materials, increase their density, and thus reduce the magnetic and dielectric losses caused by pores, simultaneously decreasing the FMR linewidth. On the other hand, the high-temperature and high-pressure environment can promote grain-boundary diffusion and uniform grain growth, improving the uniformity of YIG materials.

Yang prepared Bi-Zn-In-Sn-doped YIG ferrite using the hot-press sintering process, which significantly reduced the porosity and FMR linewidth [18]. Yang and his research team improved the magnetic and dielectric properties of BiIn-YIG ferrite and narrowed its ferromagnetic resonance linewidth through co-doping with Ca^2+^ and Sn^4+^ [19]. Zhou’s research results indicate that the co-doping of Cu^2+^-Sn^4+^ ions can enhance the saturation magnetization and reduce the FMR linewidth [20]. This can be ascribed to the low magnetocrystalline anisotropy of the two non-magnetic metal ions, Cu^2+^ and Sn^4+^. Moreover, the sample achieved optimal performance under the conditions of a doping concentration of 0.1, a sintering temperature of 1350 °C, and a holding time of 360 min. To further improve its performance, in this study, the solid-phase method was combined with the hot-press sintering process to prepare a series of Cu^2+^-Sn^4+^ co-doped YIG ferrites with different hot-press sintering temperatures, and their chemical formula is Y_3_Fe_4.8_Cu_0.1_Sn_0.1_O_12_. This study innovatively introduces the hot-press sintering process into the Cu^2+^-Sn^4+^ binary doping system of YIG ferrites, achieving multi-dimensional performance enhancement through the synergistic effect of hot-press technology and ion doping. It deeply analyzes the impact of the hot-press sintering process on the microstructure of YIG ferrite and explores the effect of the hot-press sintering temperature on its magnetic properties, aiming to find the most suitable hot-press sintering temperature.

## 2. Experiments

In this experiment, Y_3_Fe_4.8_Cu_0.1_Sn_0.1_O_12_ ferrite was prepared by the conventional high-temperature solid-phase method. Firstly, high-purity raw material powders, including Y_2_O_3_ (99.9%), Fe_2_O_3_ (99.5%), CuO (99%), and SnO_2_ (99.5%), were precisely weighed according to the stoichiometric ratio. The raw materials were placed in a ball-milling tank, and anhydrous ethanol was used as the ball-milling medium. The planetary ball mill was used to mill at a speed of 250 revolutions per minute for 12 h. After the ball-milling was completed, the obtained slurry was transferred to a drying oven for drying at 60 °C. After drying, the material was ground into powder and placed in a crucible. It was heated in a muffle furnace at a rate of 4 °C/min to 1200 °C, followed by pre-sintering at this temperature for 6 h to complete the preliminary solid-state reaction. Subsequently, the powder was put into the ball-milling tank again, and anhydrous ethanol was added as a lubricant for secondary ball-milling for 12 h. After drying, the ball-milled powder was uniformly blended with 10 wt% polyvinyl alcohol (PVA) binder and 5 wt% deionized water. Granulation was carried out under a pressure of 12 MPa, and then it was crushed. After screening with an 80–120-mesh sieve, the intermediate-layer powder was collected and compacted into a disk-shaped green body under a pressure of 4 MPa. The green body was placed in a muffle furnace for de-gumming treatment and then put into a hot-press mold. Since the sample melted at 1300 °C in a vacuum environment, the hot-press sintering temperatures were set at 1200 °C and 1250 °C, the holding time was 90 min, and the pressure was 1.5 t.

The density of the samples was measured using the Archimedes method. Phase and crystal structure analyses of the samples were performed using an X-ray diffractometer (XRD, Rigaku Cu, Rigaku Corporation, Tokyo, Japan) with CuKα radiation, where the measurements were performed over a 2θ range of 20° to 80°. A scanning electron microscope (SEM, JEOL JSM 7800F, JEOL, Tokyo, Japan) was used for high-resolution imaging of the surface and cross-section of the samples to clearly observe the microstructural characteristics of the samples, including grain size, morphology, and grain boundaries. A Raman spectrometer was used to obtain the molecular vibration information and lattice structure data of the samples. An energy-dispersive spectrometer (EDS) was employed to characterize the distribution of various elements in the samples. A vibrating-sample magnetometer (VSM, Model 3105, East Changing Technologies, Nanjing, China) was used to measure the hysteresis loop. Prior to dielectric measurement, the surface of the small piece samples was plated with silver and then fired again. Using an Agilent E4990A impedance analyzer (Agilent, Santa Clara, CA, USA), the dielectric properties of the samples were measured in the frequency range of 200 kHz to 1 MHz. The samples were placed between parallel-plate electrodes, and voltages at different frequencies were applied to record the variations in impedance and phase shift for calculating the real and imaginary parts of the dielectric constant. The FMR linewidth was measured using an Agilent N5227A vector network analyzer (Agilent, Santa Clara, CA, USA) at 9.27 GHz with the polished spherical sample placed in a TE_10_-mode rectangular waveguide resonator, where a swept magnetic field perpendicular to the waveguide axis was applied to detect the resonance via transmission coefficient variation and extract the linewidth from the full width at half maximum (FWHM) of the resonance peak.

## 3. Results and Discussion

**Figure 1:**
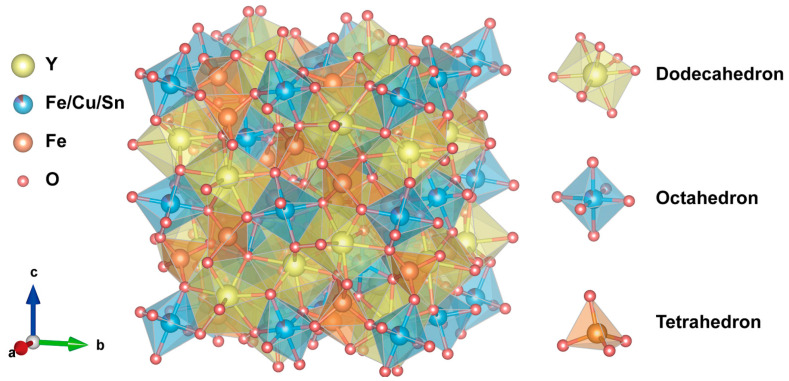
The crystal structure diagram of the Y_3_Fe_4.8_Cu_0.1_Sn_0.1_O_12_.

Figure 1 presents the crystal structure of Y_3_Fe_4.8_Cu_0.1_Sn_0.1_O_12_. Yttrium iron garnet (YIG) crystallizes in a cubic system with the space group Ia-3. A single unit cell comprises three sublattices—dodecahedral, octahedral, and tetrahedral—with specific ion arrangements. Given the constrained space between tetrahedral and octahedral sites, ions with larger radii tend to substitute Y^3+^ ions in the dodecahedral (c) sites. Ions with radii ranging from 0.6Å to 0.8Å typically occupy octahedral (a) sites, while non-magnetic ions with radii smaller than 0.6 Å are generally found in tetrahedral (d) sites. Consequently, Cu^2+^ (0.73Å) and Sn^4+^ (0.69Å) ions enter the octahedral sites, replacing the original Fe^3+^ ions.

**Figure 2:**
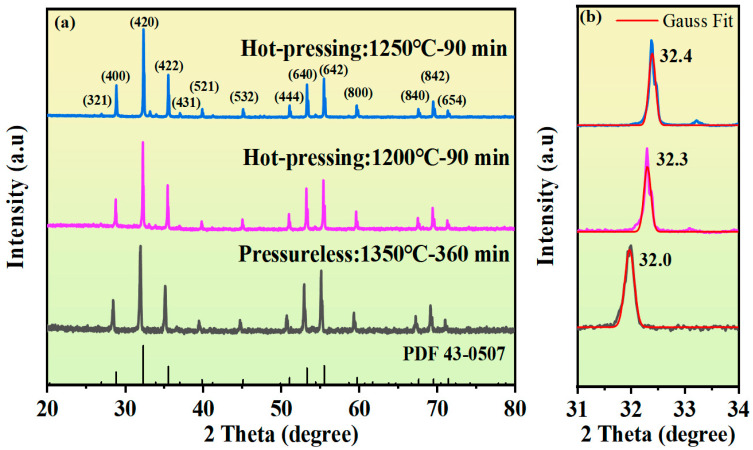
(**a**) XRD diffraction patterns of the Y_3_Fe_4.8_Cu_0.1_Sn_0.1_O_12_ ferrite under pressureless sintering and hot-press sintering, and the cubic PDF card of Y_3_Fe_5_O_12_; (**b**) Magnified view of the (420) peak.

Figure 2a presents the XRD patterns of the samples under the conditions of hot-press sintering at 1200 °C and 1250 °C for 90 min and the XRD pattern of the pressureless sintered sample. Comparison shows that the diffraction peaks of all samples are highly consistent with the standard PDF card of YIG (PDF43-0507), indicating that all samples are of a typical garnet phase structure, and that the doping of Cu^2+^ and Sn^4+^ ions has not introduced impurity phases. The enlarged view of the (420) peak is shown in Figure 2b. Compared with the pressureless sintered sample, the diffraction peak of the hot-press-sintered sample shows an obvious blue shift, indicating that hot-press sintering can reduce the lattice constant of the YIG sample. A further comparison of samples with different hot-press temperatures shows that the 420 peak of the 1250 °C hot-press sample shifts to a higher angle compared with the 1200 °C hot-press sample. This phenomenon indicates that increasing the hot-press temperature can further reduce the lattice constant of YIG ceramics.

**Figure 3:**
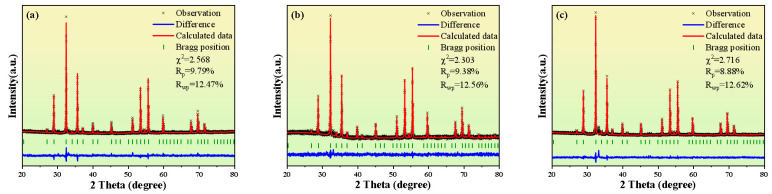
XRD refinement diagrams of Y_3_Fe_4.8_Cu_0.1_Sn_0.1_O_12_ ferrite under pressureless sintering and hot-press sintering. (**a**) Pressureless; (**b**) Hot pressing at 1200 °C; (**c**) Hot pressing at 1250 °C.

To analyze the crystal structure parameters of the ferrite more accurately, the XRD data were refined using GSAS-II, and the refinement results are shown in Figure 3 and Table 1. Usually, the reliability of the refinement results is measured by the ***χ^2^*** and ***Rwp*** values [21]. When ***χ^2^*** is close to 1 and the ***R_p_*** and ***R_wp_*** values are low, this indicates that the discrepancy between the experimental data and the calculated data is small. In this study, both indicators are within a reasonable range, indicating that the refinement results can accurately characterize the crystal structure characteristics of the samples.

**Figure 4:**
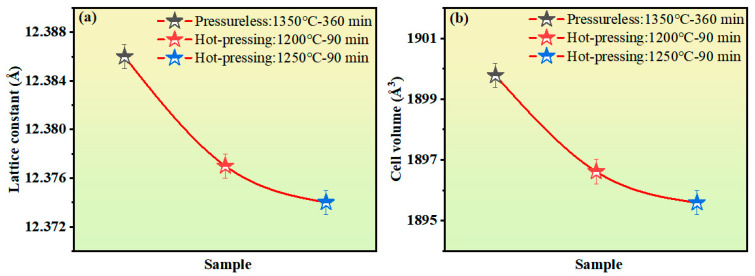
(**a**) Diagram of the variation in the lattice constant of Y_3_Fe_4.8_Cu_0.1_Sn_0.1_O_12_ ferrite. (**b**) Diagram of the variation in the unit cell volume of Y_3_Fe_4.8_Cu_0.1_Sn_0.1_O_12_ ferrite ceramics.

The lattice constant and unit-cell volume of the YIG samples can be obtained from the XRD refinement results, and their change trends are shown in Figure 4. The lattice constant and unit-cell volume of the hot-press-sintered samples are significantly smaller than those of the pressureless sintered samples. This difference can be attributed to the reduction in the distance between ions in the sample under applied pressure. A further comparison of samples with different hot-press temperatures shows that the lattice constant and unit-cell volume decrease with the increasing hot-press temperature, which is corroborated by the blue-shift phenomenon of the diffraction peak shown in Figure 2b [22]. This is because the increase in high-temperature sintering temperature will cause Cu ions to precipitate due to exceeding their solubility threshold. Owing to the larger ionic radius of Cu^2+^ (0.73Å) compared to Fe^3+^ (0.64Å), as the Cu ions precipitate, the content of Cu^2+^ in the YIG sample decreases, resulting in a decrease in the lattice constant and unit-cell volume.

**Figure 5:**
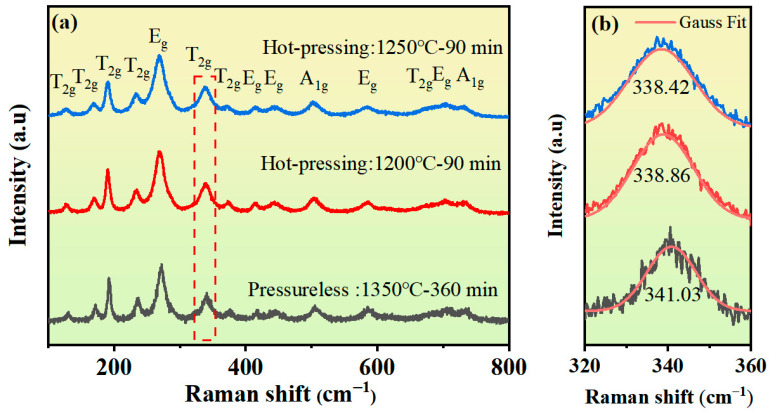
(**a**) Raman spectra of Y_3_Fe_4.8_Cu_0.1_Sn_0.1_O_12_ ferrite under pressureless sintering and hot-press sintering; (**b**) Magnified spectra of the T_2g_ peak.

Figure 5 presents the Raman spectra of the samples under different sintering conditions. A total of 14 peaks are clearly visible in the 100-800 cm^−1^ wavenumber range. Among them, the peaks located at 127, 169, 191, 233, 338, 371, and 674 cm^−1^ belong to the T_2g_ mode; the Raman peaks at 269, 415, 443, 585, and 705 cm^−1^ belong to the E_g_ mode; and the Raman peaks at 504 and 732 cm^−1^ belong to the A_1g_ mode. The Raman peaks of the samples are consistent with the characteristic absorption peaks of YIG, and no impurity peaks appear, indicating that the samples have high purity [23,24].

In the garnet lattice, the peaks between 300 and 800 cm^−1^ are primarily related to the stretching and bending of the Fe-O bonds in the tetrahedron [25,26,27]. From the T_2g_ peak spectrum shown in Figure 5b, it can be found that compared with the theoretical value of the T_2g_ peak of pure YIG (342.78 cm^−1^), the T_2g_ peak of the samples shifts obviously to the left [20]. This is because the ionic radii of Cu^2+^ and Sn^4+^ are both larger than that of Fe^3+^, and Cu^2+^ and Sn^4+^ ions successfully enter the octahedral sites, which changes the Fe-O-Fe bond length and bond angle, thereby affecting the superexchange interaction. Additionally, the Raman peak of the hot-press-sintered sample located at 340 cm^−1^ shows an obvious red shift compared with the pressureless-sintered sample, and as the sintering temperature increases, the Raman peak continues to shift to the left. This is because the Fe-O-Fe bond length becomes shorter under the action of pressure. The increase in sintering temperature causes a reduction in the content of Cu^2+^ ions with larger ionic radii, which in turn further shortens the Fe-O-Fe bond length and increases the bond angle.

**Figure 6:**
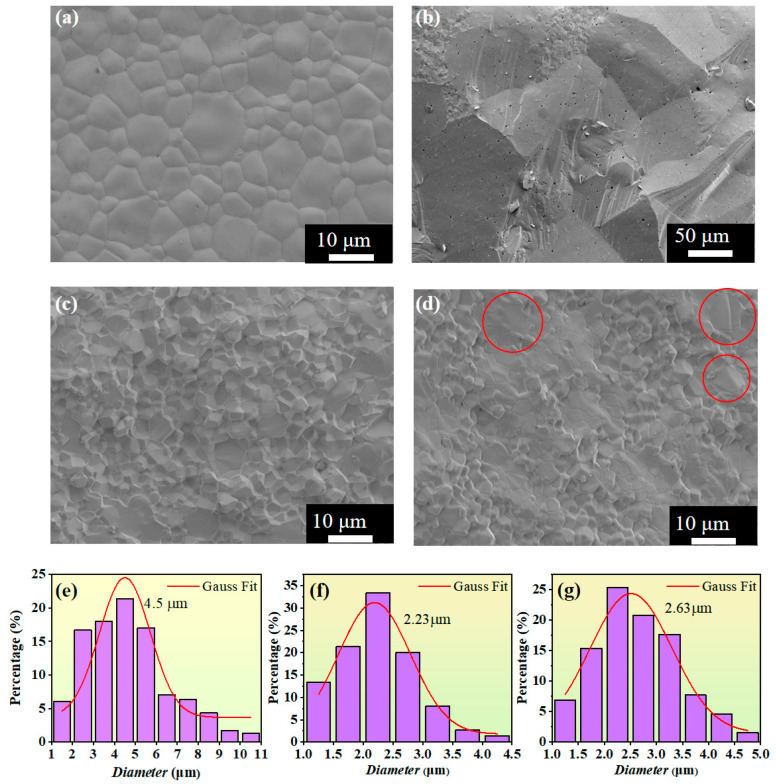
(**a**,**b**) SEM surface and cross-sectional images of Y_3_Fe_4.8_Cu_0.1_Sn_0.1_O_12_ ferrite under pressureless sintering; (**c**) SEM cross-sectional image of Y_3_Fe_4.8_Cu_0.1_Sn_0.1_O_12_ ferrite under hot pressing at 1200 °C; (**d**) SEM cross-sectional image of Y_3_Fe_4.8_Cu_0.1_Sn_0.1_O_12_ ferrite under hot pressing at 1250 °C, with large grains circled by red circles; (**e**) Particle size distribution diagram of Y_3_Fe_4.8_Cu_0.1_Sn_0.1_O_12_ ferrite under pressureless sintering and hot pressing; (**f**) Particle size distribution diagram of Y_3_Fe_4.8_Cu_0.1_Sn_0.1_O_12_ ferrite under hot pressing at 1200 °C; (**g**) Particle size distribution diagram of Y_3_Fe_4.8_Cu_0.1_Sn_0.1_O_12_ ferrite under hot pressing at 1250 °C.

Figure 6 shows the scanning electron microscope images and particle size distribution diagrams of the ferrite samples under different sintering conditions. The grains of all samples show high-density characteristics, with no obvious pores, and the grain shape is a regular polyhedron, indicating that the ions are fully diffused and regularly arranged, and the samples are fully sintered. The reason why Figure 6c,d look completely different to Figure 6b is as follows: to obtain densified YIG, pressureless sintering was carried out at 1350 °C, which led to a large grain size and a distinct large-grain morphology. Both samples (c) and (d) were prepared through hot-press sintering, with their sintering temperatures reduced by 150 °C and 100 °C, respectively, compared to the pressureless sintering temperature. As a result, not only were dense grains obtained, but the grain size was also refined. Therefore, (c) and (d) exhibit different morphologies compared to (b).

The density of the hot-press-sintered sample was measured to be 5.60 g/cm^3^ by the Archimedes method, which is much higher than that of the pressureless sintered sample (5.42 g/cm^3^). This is mainly because the 1.5 t pressure applied during the hot-press sintering process promotes the plastic deformation of the material, the mutual displacement and filling of the particles, and the extrusion of the pores, thereby enhancing the bonding strength between the particles and reducing the porosity. At the same time, the high-temperature and high-pressure environment is conducive to the diffusion and escape of gas, and will promote the recrystallization and adjustment of the grains, further suppressing the generation of pores. The lower porosity leads to the hot-press-sintered sample having a higher density.

From the particle size distribution shown in Figure 6e–g, it can be seen that the average grain size of the samples obtained by hot-press sintering is significantly smaller than that of pressureless sintering, confirming the promoting effect of the hot-press process on grain recrystallization. As the sintering temperature rises from 1200 °C to 1250 °C, the average grain size grows from 2.21 μm to 2.63 μm, indicating that the increase in the sintering temperature significantly promotes the diffusion and migration of the grain boundaries, resulting in an increase in the grain size [28,29]. In the circled areas of Figure 6d, it can be observed that some grains undergo secondary growth with increased size. This is because the higher sintering temperature enhances the thermodynamic driving force for atomic diffusion, enabling the preferential grains (formed due to local differences or defects) to consume surrounding smaller grains more rapidly, thereby inducing secondary growth.

**Figure 7:**
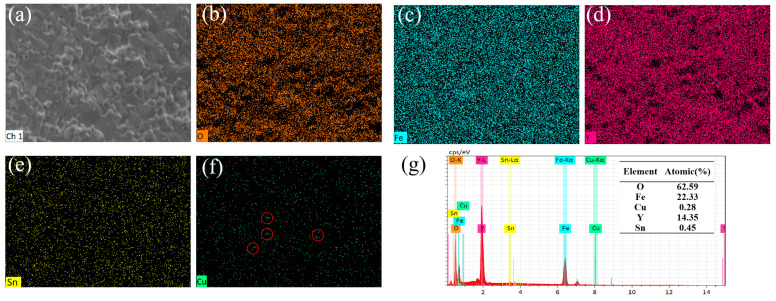
EDS spectrum of Y_3_Fe_4.8_Cu_0.1_Sn_0.1_O_12_ ferrite obtained by hot pressing at 1200 °C. (**a**) SEM image; (**b**–**f**) Element distribution diagrams of O, Fe, Y, Sn and Cu; (**g**) EDS energy spectrum.

To characterize the element composition and distribution of the YIG ferrite samples, EDS analysis was carried out on the Y_3_Fe_4.8_Cu_0.1_Sn_0.1_O_12_ sample prepared by hot-press sintering at 1200 °C. From the element distribution diagrams in Figure 7b–e, it can be seen that the elements O, Fe, Y and Sn are uniformly distributed in the sample. However, it can be found from the circled part in Figure 7f that Cu will precipitate under hot-press sintering, which is consistent with the XRD refinement results. The EDS energy spectrum data in Figure 7g show typical characteristic energy peaks for each element and there are no obvious energy peaks related to impurities. The data presented in the table of Figure 7g indicate that the atomic ratios of these elements are close to the theoretical values.

**Figure 8:**
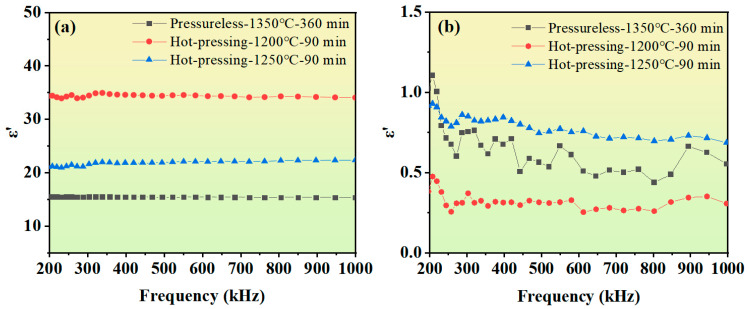
Dielectric properties of Y_3_Fe_4.8_Cu_0.1_Sn_0.1_O_12_ ferrite under pressureless sintering and hot-press sintering. (**a**) Diagram of the variation in the real part of the dielectric constant with frequency. (**b**) Diagram of the variation in the imaginary part of the dielectric constant with frequency.

Figure 8a shows the real part (***ε′***) of the complex dielectric constant of the samples in the frequency range from 200 kHz to 10 MHz, which characterizes the material’s ability to store electrical energy. The ***ε′*** decreases first and then tends to be stable with the change in frequency, and this variation law can be explained by Koop’s theory [30]. The theory points out that dielectric materials have a heterogeneous two-layer structure composed of “grains—grain boundaries”: the shell phase is a conductor (grains), and the core phase is an insulator (grain boundaries). Grain boundaries play a dominant role at lower frequencies, while grains play a dominant role at higher frequencies. Specifically, at low frequencies, charges have enough time to accumulate, forming space charge polarization, so the value of ***ε′*** is relatively high, and the dielectric loss (***ε″***) is also high due to the resistance to charge migration caused by the lattice structure. At high frequencies, charges cannot respond to the change in electric field in time, and space charge polarization disappears. At this time, ***ε′*** is mainly dominated by grains, and its polarization mainly depends on the ionic polarization inside the grains. This kind of polarization has a more stable response to high frequencies, so the dielectric constant tends to be constant. The dielectric constant is positively correlated with the polarizability rate (***χ_e_***), and their relationship can be expressed by Equation (1) [5].

(1) $$\varepsilon =1+\chi_{e}$$

Existing studies have shown that the polarization of ferrite is independent of the magnitude of the applied electric field, but decreases with the increase in the applied electric field frequency [31,32]. The ***ε′*** of YIG ferrite under hot-press sintering conditions at 1200 °C and 1250 °C is significantly higher than that of pressureless sintered samples. Among them, the ***ε′*** of the sample hot-pressed at 1200 °C reaches a maximum value of 34 at 1 MHz, which is higher compared to some similar studies [33,34]. The hot-press sintering process, as observed from the SEM results, refines the grain size and increases the number and total area of grain boundaries, thereby improving the energy storage capacity of the material.

The imaginary part (***ε″***) of the complex dielectric constant of the samples is shown in Figure 8b, which represents the electric field energy dissipation of the material. Notably, the ***ε″*** value of the sample hot-pressed at 1200 °C is the lowest, remaining below 0.5 within the frequency range of 200 kHz to 1 MHz, indicating the minimum electric field energy loss of the sample. Similar to the real part, the variation in ε***″*** with frequency also conforms to Koop’s theory. The dielectric loss of polycrystalline ferrites mainly stems from two factors: impurities and defects in the ferrite structure, and electron transitions between Fe^2+^ and Fe^3+^ ions. During the high-temperature sintering process, the formation of oxygen vacancies is inevitable. To balance the positive charges introduced by these oxygen vacancies, part of the Fe^3+^ ions in the ferrite are converted into Fe^2+^ ions. In addition, the lattice structure changes caused by the hot-press sintering process lead to variations in ***ε″***.

**Figure 9:**
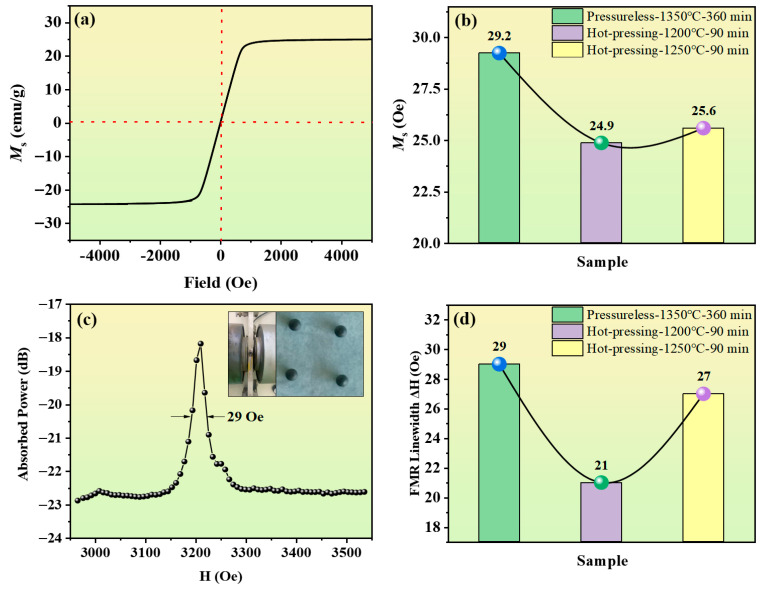
Magnetic properties of Y_3_Fe_4.8_Cu_0.1_Sn_0.1_O_12_ ferrite under pressureless sintering and hot-press sintering. (**a**) Hysteresis loop of Y_3_Fe_4.8_Cu_0.1_Sn_0.1_O_12_ ferrite under hot pressing at 1200 °C with a holding time of 90 min. (**b**) Saturation magnetization of Y_3_Fe_4.8_Cu_0.1_Sn_0.1_O_12_ ferrite under pressureless sintering and hot-press sintering. (**c**) FMR spectra of the Y_3_Fe_4.8_Cu_0.1_Sn_0.1_O_12_ ferrite under pressureless sintering, with the inset showing the FMR test sample and sample holder. (**d**) The FMR linewidth of Y_3_Fe_4.8_Cu_0.1_Sn_0.1_O_12_ under pressureless sintering and hot-press sintering.

Figure 9a shows the hysteresis loop of the Y_3_Fe_4.8_Cu_0.1_Sn_0.1_O_12_ ferrite under the hot-press sintering condition at 1200 °C (holding for 90 min). The results show that as the external magnetic field increased, the magnetization of the sample saturated rapidly. Notably, all samples in this study exhibit very low coercivity and show significant soft magnetic properties [35,36,37].

Figure 9b presents the saturation magnetization (***Ms***) of ferrites under different sintering conditions. Notably, the ***Ms*** of the pressureless sintered samples is significantly higher than that of the hot-pressed sintered samples. As the hot-press sintering temperature increases, ***Ms*** shows an increasing trend, but overall it remains lower than the level of pressureless sintering. This phenomenon can be attributed to the grain refinement caused by hot-press sintering. Smaller grains lead to a reduction in the size of magnetic domains and an increase in their number, and the magnetic domain walls also increase accordingly, thus intensifying the energy dissipation [38,39]. More energy loss results in a reduction in the overall magnetization of the material. Additionally, the use of graphite molds during hot-press sintering may introduce the carbonization of ferrites, creating oxygen defects and further contributing to the decrease in ***Ms*** [40].

To eliminate the influence of demagnetizing factors caused by shape anisotropy, the samples were polished into small spheres for FMR testing, as shown in Figure 9c, where the FMR linewidth of the pressureless sintered sample is 29 Oe. Figure 9d shows the changes in the FMR linewidth (**Δ*H***) under different sintering conditions. It can be observed that the FMR linewidth of the hot-press-sintered samples is significantly smaller than that of the pressureless sintered samples. Among them, the **Δ*H*** of the sample sintered by hot pressing at 1200 °C reaches a minimum value of 21 Oe, which is far lower than the theoretical value of 50 Oe for pure YIG and the results of other existing studies on conventional pressureless sintering [41,42,43]. According to Equation (2), the ***ΔH*** of YIG ferrite consists of the intrinsic linewidth (**Δ*H_i_***), the anisotropy field broadening (**Δ*H_a_***), the porosity broadening (**Δ*H_p_***), the incomplete solid-state reaction broadening (**Δ*H_inc_***), and the surface pit broadening (**Δ*H_sur_***) [44]. Combined with the SEM analysis, it can be seen that the hot-press sintering process substantially reduces the porosity of the ferrite samples, making the **Δ*H_p_*** of the hot-press-sintered samples much smaller than that of the pressureless sintered samples. According to Equation (3), **Δ*H_a_*** is negatively correlated with ***M_s_*** [45,46]. **Δ*H_inc_*** is mainly caused by the chemical composition inhomogeneity and magnetic inhomogeneity resulting from incomplete solid-state reactions. The XRD analysis results show that no impurity phases appear in the samples, so **Δ*H_inc_*** is basically constant. In addition, since the polished balls used for testing have a smooth surface, **Δ*H_sur_*** can be ignored. Thus, the significant change in the FMR linewidth can be attributed to the effects of hot-press sintering on porosity and ***Ms***, which in turn influence the anisotropy field broadening and porosity broadening.

(2) $$\Delta H={\Delta H}_{i}+{\Delta H}_{a}+{\Delta H}_{p}+{\Delta H}_{inc}+{\Delta H}_{sur}$$

(3) $${\Delta H}_{a}\approx 2.07{\left(\frac{K_{1}}{M_{s}}\right)}^{2}/M_{s}$$

In subsequent studies, other ions (such as rare earth ions like Gd^3+^ and Yb^3+^) can be introduced based on the Cu^2+^-Sn^4+^ co-doping system to systematically investigate the synergistic regulation of multi-element doping on the ferromagnetic resonance linewidth. Rare earth ions with larger ionic radii will occupy the dodecahedral sites in the garnet structure, thereby altering the lattice structure and further influencing the magnetic and dielectric properties. Additionally, as the ferrite prepared by the solid-phase reaction method in this study has a relatively large particle size, new synthesis methods such as the sol–gel method can be adopted in the future, combined with multi-element doping and hot-press sintering process to further optimize the magnetic and dielectric properties of YIG ferrite.

## 4. Conclusions

This study investigated the effects of the hot-press sintering temperature on the dielectric and magnetic properties of YIG materials. In the experiment, a series of Y_3_Fe_4.8_Cu_0.1_Sn_0.1_O_12_ samples under different sintering conditions were prepared by combining the solid-phase method with the hot-press sintering process. The influence and mechanism of the hot-press sintering process on the microstructure dielectric properties and magnetic properties of YIG were deeply explored, and the following conclusions were drawn: The hot-press sintering process has a significant effect on optimizing the microstructure of the material. It substantially reduces the porosity of the material and significantly improved the density. The YIG sample under the hot-press condition at 1200 °C has the highest dielectric constant (34), the lowest dielectric loss and the smallest FMR linewidth (21 Oe). This study fully confirms the effectiveness of the hot-press sintering process in improving the microstructure of YIG materials and their dielectric and magnetic properties.

## Figures and Tables

**Table 1:** Crystal refinement data of Y_3_Fe_4.8_Cu_0.1_Sn_0.1_O_12_ ferrites under different sintering conditions.

Sintering Method	Pressureless Sintering	Hot-Press Sintering	Hot-Press Sintering
Temperature (°C)	1350	1200	1250
Holding Time (min)	360	90	90
Lattice Constant (a = b = c, Å)	12.387	12.377	12.375
R_p_ (%)	9.79	9.38	8.88
R_wp_ (%)	12.47	12.56	12.62
χ^2^	2.568	2.303	2.716
Lattice constant	12.385	12.377	12.374
Cell Volume (Å^3^)	1899.78	1896.61	1895.59

## Data Availability

The original contributions presented in this study are included in the article. Further inquiries can be directed to the corresponding author.
